# A multiplex assay to detect mosquito species, bloodmeal host source and *Plasmodium* in malaria vectors using Nanopore amplicon sequencing

**DOI:** 10.1111/mve.70055

**Published:** 2026-02-12

**Authors:** E. Abby Rogers, Dieunel Derilus, Christopher Sandi, Lisa Reimer, Audrey Lenhart, Lucy Mackenzie Impoinvil

**Affiliations:** ^1^ Entomology Branch, Division of Parasitic Diseases and Malaria National Center for Emerging and Zoonotic Infectious Diseases, Centers for Disease Control and Prevention Atlanta Georgia USA; ^2^ Department of Vector Biology Liverpool School of Tropical Medicine, Pembroke Place Liverpool UK

**Keywords:** amplicon sequencing, MinION, species identification, vector surveillance

## Abstract

Multiple species of *Anopheles* mosquitoes transmit malaria‐causing *Plasmodium* around the world. Molecular methods are often employed to confirm vector species, detect parasites and determine bloodmeal host sources; these assays are often performed separately and can be time‐consuming and expensive. However, in this study, we show that the Oxford Nanopore Technologies (ONT) MinION Sequencer offers a cost‐effective and efficient alternative to accurately identify mosquito species, host bloodmeal sources and detect parasites simultaneously in malaria vectors. We sequenced 150 insectary‐reared mosquitoes representing nine species and 150 blood‐fed mosquitoes with one of five vertebrate blood sources. We also analysed the presence of *Plasmodium falciparum* (Welch, 1897) in 40 infected mosquito samples. A final combined assay integrated all three previously optimized assays into a single sequencing run, demonstrating the high‐throughput capability of the Nanopore sequencing platform. This run included 32 samples for each targeted amplicon, totalling 96 samples. For comparison, we sequenced all samples using a standard Sanger sequencing protocol. Our results showed that the MinION sequencing platform accurately identified all nine mosquito species, five different bloodmeal hosts from the blood‐fed mosquitoes up to 48 h post blood feed, and detected *P. falciparum* in the 40 positive controls. The sequencing results obtained using the MinION platform exhibited high concordance with those from standard Sanger sequencing, as demonstrated by comparable similarity scores and correct mosquito species identification. This demonstrates that our MinION sequencing and analysis protocol offers a novel, highly precise, cost‐effective solution for combined mosquito species identification, bloodmeal analysis and parasite detection.

## Graphical abstract

- Insectary‐reared mosquitoes were simultaneously assessed for species identification, bloodmeal source analysis and *Plasmodium* detection, using a single multiplexed sequencing run on the MinION sequencing platform.
- The method was applied to 150 insectary‐reared mosquitoes, representing nine species blood‐fed with five vertebrate hosts and 40 samples for parasite detection.
- Results show high concordance with standard Sanger sequencing, making MinION a viable tool for sequencing in resource‐limited areas.

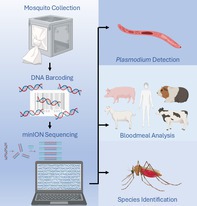

## INTRODUCTION

Malaria is a central focus of the World Health Organization's (WHO) global disease eradication efforts due to its significant impact on public health (World Health Organization, 2015). Anopheline (Diptera:Culicidae) mosquitoes transmit the six malaria‐causing parasites that infect humans, with *Plasmodium falciparum* (Welch, 1897; Haemosporida: Plasmodiidae) causing the most severe symptoms (Milner, 2018). Despite important reductions in the global burden of malaria, there is still significant morbidity and mortality across the globe. To address this burden, it is imperative to accelerate innovation in laboratory analyses of malaria vectors. Mosquito vectors have shown significant adaptability to currently employed control mechanisms, including insecticide resistance via reduced target site sensitivity or metabolic detoxification (Bamou et al., 2021). As vectors adapt, the methods for controlling them must advance as well. These advancements rely on entomological surveillance data, and novel strategies for rapid characterization of vectors are needed, especially in areas of high malaria burden. Accurate mosquito species identification, understanding blood‐feeding behaviours and parasite detection are crucial in understanding malaria transmission risk and are critical to informing which vector control interventions and strategies to deploy.

Identifying morphologically similar mosquito species can be particularly challenging due to subtle physical differences that may be difficult to distinguish under standard microscopy. Typical methods of identification involve the use of taxonomic keys, which require the expertise of well‐trained personnel. Using taxonomic keys is prone to human errors; some species are morphologically indistinguishable, and identification requires the use of intact mosquitoes that are not damaged or discoloured (Jourdain et al., 2018). Mosquito samples may lose key features such as scales, wings or legs during trapping and collection as part of routine surveillance (Garjito et al., 2021).

Molecular identification using PCR‐RFLP (restriction fragment length polymorphism) is a widely used method to distinguish between morphologically indistinguishable species. This method uses ribosomal DNA, such as the internal transcribed spacer 2 (ITS2) region, for species determination (Collins & Paskewitz, 1996), and cytochrome c oxidase, subunit 1 (CO1) gene for differentiation between mosquito species through PCR‐based methods (Escobar et al., 2020). Amplification of the CO1 gene is primarily used as a standard complementary method to ITS2 amplification when differentiating between closely related species during a PCR‐based identification method (Rach et al., 2017). However, this complementary method is costly and time‐consuming, creating challenges for its use in routine screening. In groups of mosquitoes with high genetic similarity, such as the *Anopheles gambiae* complex, the ITS2 region can show more variation, making it valuable for identifying closely related species on its own (Collins & Paskewitz., 1996).

Furthermore, studies have reported misidentification of the *Anopheles gambiae* complex and *An. funestus* (Giles, 1900) group using multiplex PCR assays (Dahan‐Moss et al., 2020; Erlank et al., 2018). Successful control of vector‐borne diseases relies on the correct identification of mosquito vectors (Mishra et al., 2021), as some morphologically identical species can vary in their biology. Misidentification can lead to the application of the wrong vector control intervention. For example, *An. arabiensis* (Patton, 1905), which is part of the *An. gambiae* complex, is more likely to rest outdoors and is not an ideal target for an intervention such as indoor residual spraying (IRS) (Githeko et al., 1994).

Along with mosquito species identification, understanding the feeding preference and behaviours of vectors is important to guide control methods (Tedrow et al., 2019). The feeding patterns of mosquitoes play a key role in the dynamics of pathogen transmission and can be influenced by both extrinsic and intrinsic factors (Fikrig & Harrington, 2021). Likewise, intake of a bloodmeal is necessary for the completion of a female's gonotrophic cycle and acts as a bridge for the transmission of the *Plasmodium* species that cause malaria. Studies have also shown that multiple host feedings can shorten *Plasmodium* incubation periods; therefore, increasing the potential for disease transmission (Shaw et al., 2020).

Due to the relatively low prevalence of pathogens in mosquitoes, a high number of individual samples need to undergo molecular testing for accurate representation of pathogen prevalence in a locality (Echeverry et al., 2017). Molecular identification of host bloodmeals and presence of *Plasmodium* are primarily achieved through standard PCR (Kumpitak et al., 2021). However, PCR‐based methods alone are limited and have the potential to report false negative results (Caixeta et al., 2014). Loop‐mediated isothermal amplification (LAMP) can detect low‐level *Plasmodium* infections in the field and offers rapid, simple diagnostics. However, it requires six to eight primers per gene, making design more complex than standard PCR, and species identification depends on selecting appropriate primers (Kamber et al., 2020). In addition, LAMP samples do not produce large amounts of DNA and may not be suitable for sequencing (Selvarajah et al., 2020).

The most accurate method for species identification is sequencing. Sanger sequencing is the gold standard due to its accuracy and reliability (Cheng et al., 2023). However, it is limited in its sequence length, maintenance cost and requires a specialized laboratory setting. Next‐generation sequencing (NGS) technologies provide an alternative to Sanger sequencing and have the potential to overcome some of the limitations of Sanger sequencing (Mardis, 2013). Nevertheless, a substantial limitation that remains is the incomplete coverage of reference databases. Only about 28%–30% of known mosquito species have COI barcodes, and roughly 12% have ITS2 sequences publicly available in GenBank, which restricts the ability to assign species names to sequences from many taxa (Moraes Zenker et al., 2024).

The Oxford Nanopore Technologies (Oxford, United Kingdom) MinION sequencing platform is a portable NGS tool that employs real‐time sequencing, unrestricted read lengths and technology adaptive to field‐based methods (Wang et al., 2021). Protocols adapted for resource‐limited or nonconventional laboratories could provide robust and efficient surveillance methods in areas with a high burden of vector‐borne disease. These portable protocols also extend laboratory independence and decrease sequencing costs, allowing for improved implementation of mosquito surveillance in areas that would otherwise be dependent on external laboratories for sample processing. Nanopore's MinION has successfully been applied in remote sites in West Africa to monitor the 2014–2015 Ebola outbreak (Quick et al., 2016) and in the Ecuadorian Chocó rainforest (Pomerantz et al., 2018). Additionally, multiplexing across a bulk of mosquito samples allows for simultaneous sequencing of different target genes, maximizing the data output per sample. MinION sequencing offers ample coverage depth to provide a de novo assembly approach of the targeted amplicons. As of now, the MinION sequencer is the only method that allows for this kind of large output in an affordable and time‐sensitive manner, with a maximum output of 400 bases per second (Wang et al., 2021). ONT has been used for detection of parasites (Girgis et al., 2023), viruses (Mirza et al., 2024) and for mosquito species identification (Hartke et al., 2022) but has not been used in multiplex assays for the simultaneous detection of host, parasite and mosquito species, specifically of the closely related species of the *An. gambiae* complex.

Here, we describe the development of a field‐applicable protocol that allows for the simultaneous assessment of mosquito species identification, bloodmeal analysis and *P. falciparum* detection using a multiplex approach through the MinION sequencing platform (Wasswa et al., 2022). This method utilizes a field‐applicable DNA extraction protocol, PCR amplification platform, PCR cleanup protocol and pooling of multiple PCR amplicons into a single MinION sequencing run. In addition to developing this MinION‐based approach, we also compare its performance to traditional Sanger sequencing across the same targets to evaluate the reliability, scalability and potential field utility of each method.

## MATERIALS AND METHODS

### Mosquito rearing

All mosquitoes were reared under standard insectary conditions (27°C ± 2°C, 80% relative humidity, light:dark cycles of 14:10 h) with access to 10% sucrose solution and blood‐fed on a Hemotek membrane feeding system (Hemotek Ltd., Blackburn, United Kingdom) with defibrinated rabbit blood. Initially, three sequencing assays were performed separately for optimization purposes, with various sample sizes: mosquito species identification (*n* = 150), host bloodmeal analysis (*n* = 150) and *P. falciparum* detection (*n* = 40). A final simultaneous assay combined all three previously optimized assays in one single sequencing run to evaluate the multiplex capacity of the Nanopore sequencing platform, with a sample size of *n* = 32 for each targeted amplicon assay, for a total of *n* = 96. There were nine species, including morphologically similar species of the *Anopheles gambiae* complex, represented in the mosquito species identification experiment. All species were supplied by Malaria Research and Reference Reagent Resource Center (MR4), BEI Resources, NIAID NIH (Manassas, VA, USA) and are categorized as follows: subgenus *Cellia*: *An. gambiae* (Patton), *An. coluzzi* (Coetzee & Wilkerson), *An. funestus* (Giles), *An. stephensi* (Liston), *An. minimus* (Theobald), *An. merus* (Donitz), *An. arabiensis* (Patton), subgenus *Nyssorhyncus*: *An. albimanus* (Wiedemann) and subgenus *Anopheles*: An. *freeborni* (Aitken). The 150 mosquitoes that were used for the blinded mosquito species identification assays were knocked down at 3–5 days old and stored at −20°C.

The *An. albimanus* Sanarate strain was used for the bloodmeal analysis sequencing assays, with a total of 150 specimens representing five time points post feeding. Adult mosquitoes were fed for 35 min using an electronically heated Hemotek (UK) membrane feeder, with one of the five host bloods: human, goat, cow, rabbit or guinea pig. Engorged females were then placed in a separate cage to be collected at the following time points: 0, 24, 48, 72, and 96 h post feeding. At each of the time points, five blood‐fed females were collected and knocked down at −20°C. This experiment was repeated three times with different sample storage conditions. One subset of samples (*n* = 50) was immediately blood‐spotted and stored at room temperature overnight, prior to DNA extraction. Bloodspots were obtained by squishing mosquito abdomens on 3 mm Whatman™ Filter Paper and dried overnight prior to DNA extraction. Another subset of samples (*n* = 50) was knocked down at −20°C and then transferred to a 2‐mL tube with 350 mg of Drierite® desiccant to be stored at room temperature. The dried samples were stored at room temperature for 2 weeks prior to DNA extraction. Lastly, a subset of samples (*n* = 50) was knocked down at −20°C and immediately submerged in 250 μL of 100% ethanol (EtOH) and left at room temperature for 1 week prior to DNA extraction. Samples were stored for varying durations to ensure complete desiccation prior to DNA extraction, with drying confirmed across all three methods by visual observation of the absence of moisture and tissue pliability.

For the *Plasmodium* detection assay, 40 *An. gambiae* females that had been fed *Plasmodium falciparum‐*spiked bloodmeals containing 0.5% mature gametocytes were used as positive controls. Additionally, 20 more uninfected, insectary‐reared *An. gambiae* were used as negative controls. Infected and uninfected individuals were knocked down and stored at −20°C prior to DNA extraction.

### DNA extraction

For the assays, DNA was derived from several sources: bloodspots (mammalian blood controls and mosquito abdomens), whole mosquitoes preserved in 100% ethanol, whole mosquitoes stored in desiccant (350‐mg Drierite®) and whole mosquitoes stored at −20°C. Whole mosquitoes stored in ethanol were carefully removed with forceps and blotted dry prior to DNA extraction. For bloodspots, whole mosquitoes were taken and pressed onto 3‐mm Whatman™ Filter Paper. The bloodspot was then dried at room temperature for 24 h and cut from the paper using sterile scissors, then submerged in extraction buffer. Genomic DNA was extracted from either whole mosquitoes or dried bloodspots using the Extracta™ DNA Prep for PCR‐Tissue Kit (QuantaBio, USA), as described in the manufacturer's protocol. The protocol calls for a 1:1 ratio addition of an extraction buffer and stabilization buffer, with a 30‐min incubation at 95°C. This kit was chosen for its compatibility with the battery‐powered miniPCR™ machine, offering a portable alternative to conventional equipment like water baths or thermal cyclers and enabling efficient DNA extraction without the need for standard laboratory infrastructure. DNA was quantified using a NanoDrop 2000 Spectrophotometer (Thermo Scientific), which operates on the principle of ultraviolet–visible spectrum absorbance. The spectrum normalization was achieved by setting the baseline absorbance to that of the Stabilization Buffer provided in the Extracta™ DNA Prep for PCR‐Tissue PCR Kit (QuantaBio, USA). For quantification, 1.0 μL of the sample's extracted DNA was used.

### PCR amplification

**FIGURE 1:**
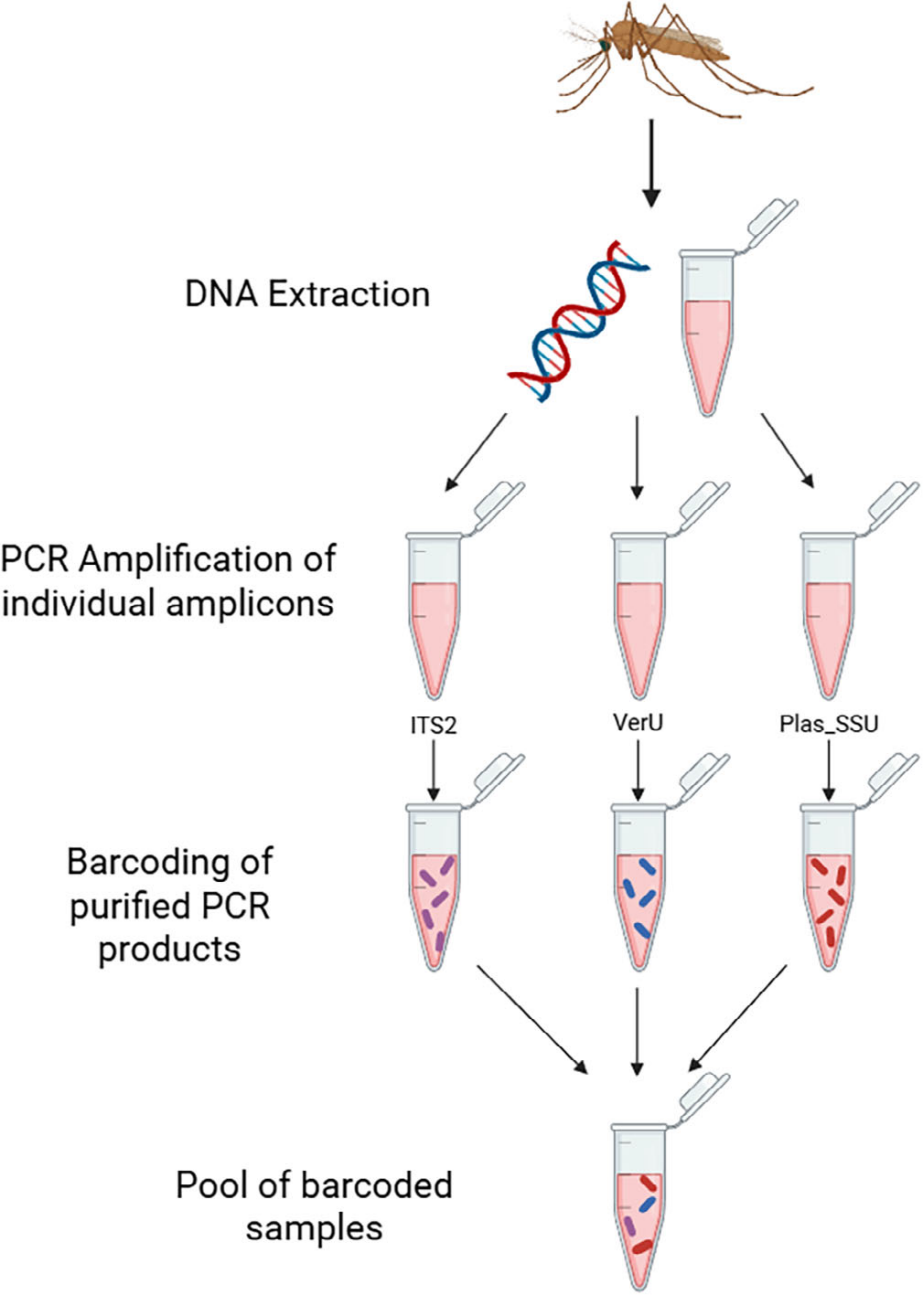
Workflow illustrating the molecular approach used to simultaneously detect mosquito species, bloodmeal source and *Plasmodium* infection. The process included DNA extraction, PCR amplification of three target amplicons and multiplexing for nanopore sequencing. DNA from individual mosquitoes was added to three 0.2‐mL thin‐walled PCR tubes containing the appropriate primer sets and master mix. Amplified products were purified, barcoded through a brief incubation and pooled for library preparation prior to sequencing.

DNA amplification was performed for each of the chosen assays (mosquito species identification, *Plasmodium* detection and bloodmeal analysis) (Figure 1). The reaction mixtures contained 12.5 μL of Accustart II PCR Supermix (QuantaBio), 1.0 μL of 10 μM forward primer, 1.0 μL of 10 μM reverse primer, 8.5 μL of nuclease‐free water and 2.0 μL of DNA. The three separate amplifications were performed simultaneously, using the following conditions: 94°C for 4 min; 35 cycles of 94°C for 1 min, 51°C for 1 min and 72°C for 1 min; and 72°C for 10 min. Water was included as a negative PCR control to ensure the absence of any contamination during the amplification process. Primer sequences can be found in Table 1. A 1% agarose gel electrophoresis was conducted using GelRed™ for visualization (Figures S1–S3). PCR products were purified with ExoSAP‐IT™ (Applied Biosystems) in accordance with the manufacturer's protocol. Purified samples were stored at −20°C prior to library preparation.

**TABLE 1:** Primer sequences used to amplify the targeted regions of genomic DNA from whole mosquitoes.

Primer name	Forward sequence 5′–3′	Reverse sequence 5′–3′	*T*_m_	Expected amplicon size	References
ITS2	TGTGAACTGCAGGACACATGAA	ATGCTTAAATTTAGGGGTAGTC	51°C	300–1000 bp	(Henry‐Halldin et al., 2011)
VerU	AAGACGAGAAGACCCYATGGA	CCTGATCCAACATMGAGGTCGTA	51°C	280 bp	(Ejiri et al., 2011)
SSU_Plas	TTCAGATGTCAGAGGTGAAATTCT	AATTAGCAGGTTAAGATCTCGTTC	51°C	422 bp	(Tedrow et al., 2019)

*Note*: ITS2 targets the Internal Transcribed Spacer 2. VerU targets the vertebrate 16S ribosomal RNA region, and SSU_Plas amplifies the small subunit ribosomal RNA of *Plasmodium* spp.

The miniPCR™ (miniPCR bio, Cambridge, MA, USA), a durable and compact thermal cycler, was used in conjunction with a traditional thermal cycler to demonstrate the portability of this protocol. Standard PCR reagents and consumables are compatible with the miniPCR™, allowing for the same PCR amplification conditions and reagents to be used with both methods. The blueGel™ visualizer (miniPCR bio, Cambridge, MA, USA) is an integrated gel electrophoresis and visualization system and was used to verify the presence of the targeted amplicon prior to sequencing, according to the manufacturer's protocol. The miniPCR™ and blueGel™ instruments are portable and can be run on battery power, utilizing either a laptop or a Bluetooth connection to a smart phone to run the instrument. Both pieces of equipment were used to improve accessibility and demonstrate the portability of these protocols and their required equipment.

### Sanger sequencing

Standard Sanger sequencing was used in conjunction with the MinION platform to compare with the amplicon‐based approach and its accuracy. All three targeted amplicon products (ITS2, 16 s and SSU rRNA) were sequenced with Sanger technology. Each 10 μL reaction included the following: 0.5 μL of Terminator Ready Reaction Mix v1.1, 2.0 μL CDC 5x Sequencing Buffer, 1.0 μL of 5 μM primer, 5.5 μL nuclease‐free water and 1.0 μL DNA template. PCR cycling conditions were: 25 cycles of 96°C for 10 s, 50°C for 5 s, 60°C for 4 min. Products were further purified using the BigDye xTerminator™ Purification Kit, according to the manufacturer's protocol. The ABI 3130xl machine (Applied Biosystems, Thermo Fisher Scientific, Waltham, MA, USA) was used for sequencing. Sequencing files were aligned using SeqMan Pro™. A Basic Local Alignment Search Tool – nucleotide (BLASTn) search was then performed on all aligned Sanger sequences to check for homology.

### MinION library preparation

MinION library preparation was conducted using the Rapid Barcoding Kit (SQK‐RBK110.96) and sequencing protocol from ONT. This kit is designed to minimize cross‐contamination and index cross‐talk through the use of unique molecular identifiers and barcodes, specifically designed to reduce misassignment during library preparation. For the 96 sample barcoded library, a subset of PCR products (*n* = 32) from each of the targeted regions (ITS2, VerU and Plas) was taken forward for multiplexed sequencing on the MinION. The concentration of the purified PCR products obtained from DNA amplification were measured using the Qubit™ dsDNA Broad Range Assay, according to the manufacturer's protocol. The target DNA concentration for each sample was between 50 and 200 ng in 9.0 μL. This volume was carried forward and barcoded using 1 μL of barcode provided in the kit. Barcoded samples were incubated at 30°C for 2 min and then at 80°C for 2 min. Tubes were then briefly cooled on ice. The 96 barcoded samples were then diluted with nuclease‐free water and pooled into a desired ratio with a total library concentration of 1000 ng (Figure 1). Library purification was performed using the SPRI beads provided in the ONT Rapid Barcoding Kit (SQK‐RBK1110.96) with a 1:1 ratio, and a 5‐min incubation at room temperature. The eluted library was then quantified using the Qubit™ dsDNA Broad Range Assay and diluted to a concentration of 50 fmol with a total volume of 12.0 μL. The entire volume of library was loaded and sequenced on a primed MinION Spot‐On Flow Cell Mk1B (R9 version) for 4–12 h, depending on library and targeted amplicon size. During each sequencing run, FAST5 output, FASTQ output and basecalling were enabled. Read filtering set the minimum quality score to 7, with the minimum barcode score of 60 bp. Sequencing with the MinION platform was performed using a commercially available laptop equipped with an Intel i7 processor, 32GB of random access memory (RAM) and a 500GB solid‐state drive (SSD).

### Basecalling and demultiplexing

In this experiment, the basecalling was performed in real‐time during sequencing using MinKNOW (provided by ONT), while the demultiplexing of the nucleotide sequences was performed in real‐time using Guppy (v6.4.6) (also provided by ONT).

### Quality control and downstream analysis

The quality of the Nanopore sequencing reads was assessed and visualized using NanoPlot 1.28.2 (De Coster et al., 2018). For species identification, the downstream analysis of the ONT reads comprised two primary steps: the generation of a consensus sequence and the subsequent BLASTn search of the consensus sequence against the relevant database. Strong BLASTn matches for each targeted region were accepted, provided the consensus was represented by more than one read. In this study, strong BLASTn matches of the consensus sequence were determined with a ≥ 97% similarity and query cover of ≥ 650, ≥ 280 and ≥ 422 bp for ITS2, VerU and SSU_Plas, respectively. To generate accurate consensus sequences from the raw sequencing datasets, we benchmarked two bioinformatics software tools, Geneious Prime (version 2022.0.2) and ONTrack (version 1.5.0) (Figure S4) (Maestri et al., 2019).

The species identification pipeline using the two respective tools is briefly described here:

Geneious prime: Passed FASTQ files were obtained from the MinKNOW software and imported into Geneious Prime. Upon importing, Oxford Nanopore was selected as the Read Technology, and all sequences were combined into a sequence list for each barcoded sample. Sequences underwent a de novo assembly within Geneious Prime. Contigs were dissolved and reassembled with medium sensitivity. Final consensus sequences were compared to the National Center for Biotechnology Information (NCBI) database using BLASTn to determine the species ID.

ONTrack: The raw demultiplexed FASTQ files produced by the MinKNOW software were concatenated and converted to FASTA format, resulting in one FASTQ and one FASTA file per sample. The configuration file provided by ONTrack (config_MinION_mobile_lab.R) was updated with the following non‐default parameters: ‘amplicon_length <‐700’ (mean amplicon length in base pairs), ‘primers_length <‐22’ (length of the PCR primers in base pairs) and ‘do_blast_flag <‐1’ (perform BLASTn search of the consensus sequence) as described by Maestri et al. (2019). The ‘ONTrack.R’ pipeline script was then employed to generate an accurate consensus sequence for each sample, which was subsequently subjected to a BLASTn search against the local NCBI database for species identification. This pipeline includes several steps including: clustering of ONT reads using VSEARCH (Rognes et al., 2016), random sampling of the read clusters using Sektq (https://github.com/lh3/seqtk), generation of the consensus draft using MAFFT (Katoh et al., 2002) and EMBOSS (Rice et al., 2000) consensus polishing using Nanopolish (Hu et al., 2021) and BLASTn search of the polished sequence for species identification (Supplemental Materials).

### Sanger sequence analysis

Reads obtained through Sanger sequencing (.ab1 files) were imported into SeqMan Pro™ 17 (Version 17.3.0). Forward and reverse sequences were concatenated and assembled using default parameters. Final consensus sequences were compared to the NCBI database using the BLASTn to determine if the correct taxonomy was found.

## RESULTS

### Assessing MinION accuracy and efficacy

**FIGURE 2:**
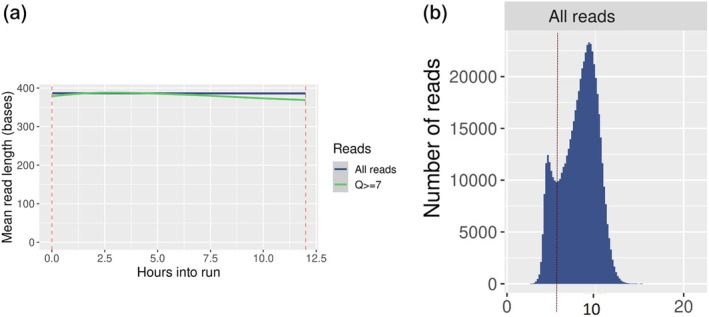
Quality control assessment of Oxford Nanopore Technologies sequencing data for the multiplexed mosquito, bloodmeal and *Plasmodium* amplicons. (a) Mean read length remained stable throughout the sequencing run, indicating consistent sequencing of the intended target amplicons. (b) Distribution of raw read quality scores (Q scores) used for downstream analysis. Only reads with a Q score ≥ 7 were retained for analysis; the red line indicates the quality cut‐off, and reads below this threshold were excluded to ensure high‐confidence sequence data.

**FIGURE 3:**
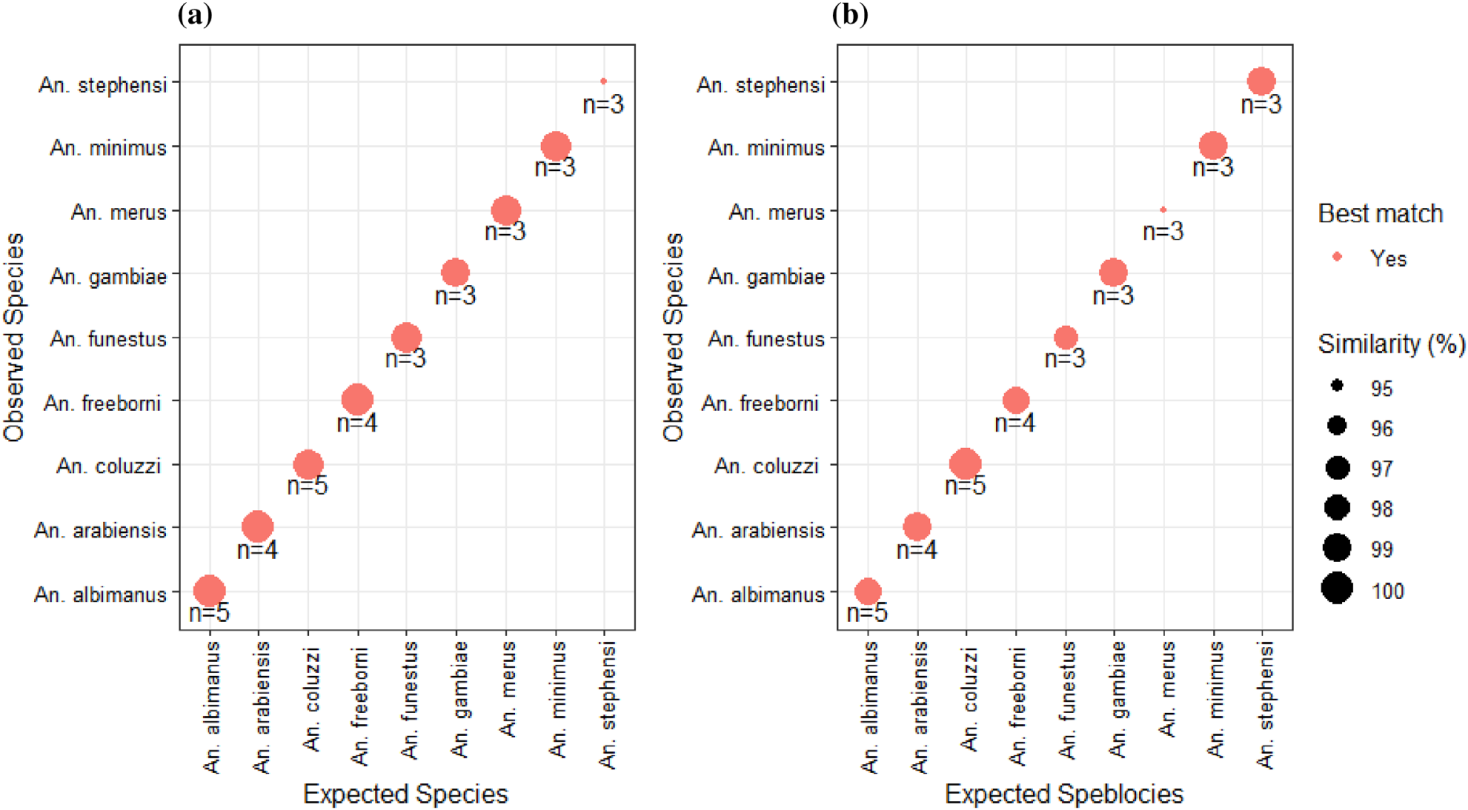
Accuracy of the multiplex nanopore sequencing assay for simultaneous detection of mosquito species, bloodmeal host and *Plasmodium* infection. Percent sequence similarity between Oxford Nanopore MinION reads and the corresponding reference sequences obtained by standard Sanger sequencing is shown for the three target amplicons: Plasmodium SSU rRNA (SSU_Plas), host bloodmeal cytochrome b (VerU) and mosquito ITS2 region (ITS2). Across all targets, MinION consensus sequences achieved an average similarity of over 96% compared to Sanger sequencing, demonstrating the high fidelity of the multiplex assay.

Sequencing of a barcoded 96‐sample library, targeting three separate amplicons, yielded a total of 586.2 k passed reads, with an average of approximately 6107 reads per sample (Table S1). To determine how differing concentrations may affect read output, the samples were grouped according to four different ranges of initial sample concentration input with their corresponding average number of reads: 0–20 ng/μl (1220 reads), 21–50 ng/μl (4326 reads), 51–100 ng/μl (4930 reads) and > 100 ng/μl (13,166 reads). For all MinION sequencing runs, real‐time output was examined to ensure the correct regions were being sequenced and the quality of the reads was acceptable. Throughout sequencing, the mean Q‐score stayed above 7, ensuring only quality reads were carried forward through data analysis (Figure 2b). The mean read length stayed consistent throughout sequencing, validating that the targeted amplicons were being sequenced (Figure 2a). Each utilized barcode was recognized during the sequencing runs. Percent similarity between Sanger and MinION sequencing, across all three targeted amplicons averaged above 96% (Figure 3).

### Mosquito species identification

A total of 150 *Anopheles* individuals representing nine species were extracted for targeted amplicon sequencing of the ITS2 region. Quantification of the extracted DNA showed a concentration range of 152 ng/μl to 222 ng/μl. PCR amplification and gel electrophoresis of the DNA confirmed that the intended ITS2 target was amplified, with expected band sizes ranging from 460 to 700 bp, dependent on the mosquito species.

**FIGURE 4:**
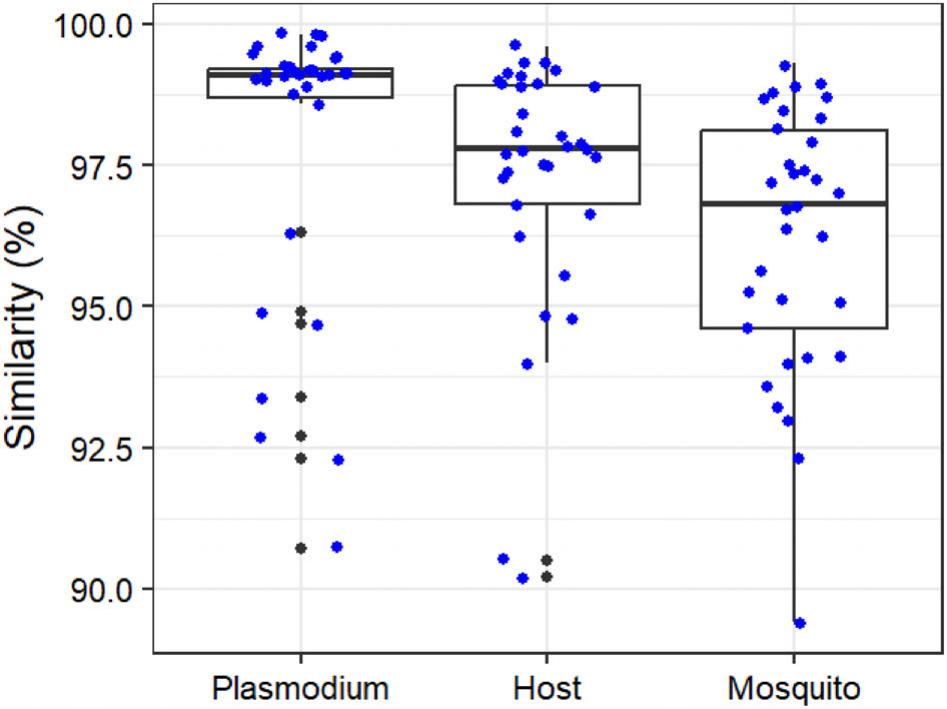
Identification of *Anopheles* species using consensus sequences from Oxford Nanopore Technologies reads. Consensus sequences were generated with the ONTrack pipeline (a) or Geneious Prime (b) and compared to reference sequences via BLASTn. The Y‐axis shows the expected species identity confirmed by Sanger sequencing, while the X‐axis represents the observed species, defined as the top BLASTn hit based on mean sequence similarity (%). Circle colour indicates identification accuracy (red = species correctly identified as expected), and circle size reflects the degree of sequence similarity between the consensus and reference sequences. This analysis demonstrates the accuracy of nanopore‐based species identification in the multiplex assay.

A total of 526.25 k reads were obtained for the sample library used for sequencing of the ITS2 region for mosquito species identification. The species of each mosquito was successfully derived from the MinION reads for all 150 samples. The BLASTn search of the consensus sequences generated through both bioinformatics analysis approaches (Geneious Prime and ONTrack) exhibited consistent results in terms of best BLASTn hit, mean percent of similarity and coverage, confirming the reproducibility and consistency of the approach for mosquito species identification. These findings were consistent with the standard Sanger sequencing results, with a 100% concordance (Figure 4), confirming the accuracy of the ONT amplicon sequencing approach presented here. Likewise, a BLASTn search using the NCBI database matched the sequenced ITS2 regions with the correct mosquito species.

### Bloodmeal analysis

**FIGURE 5:**
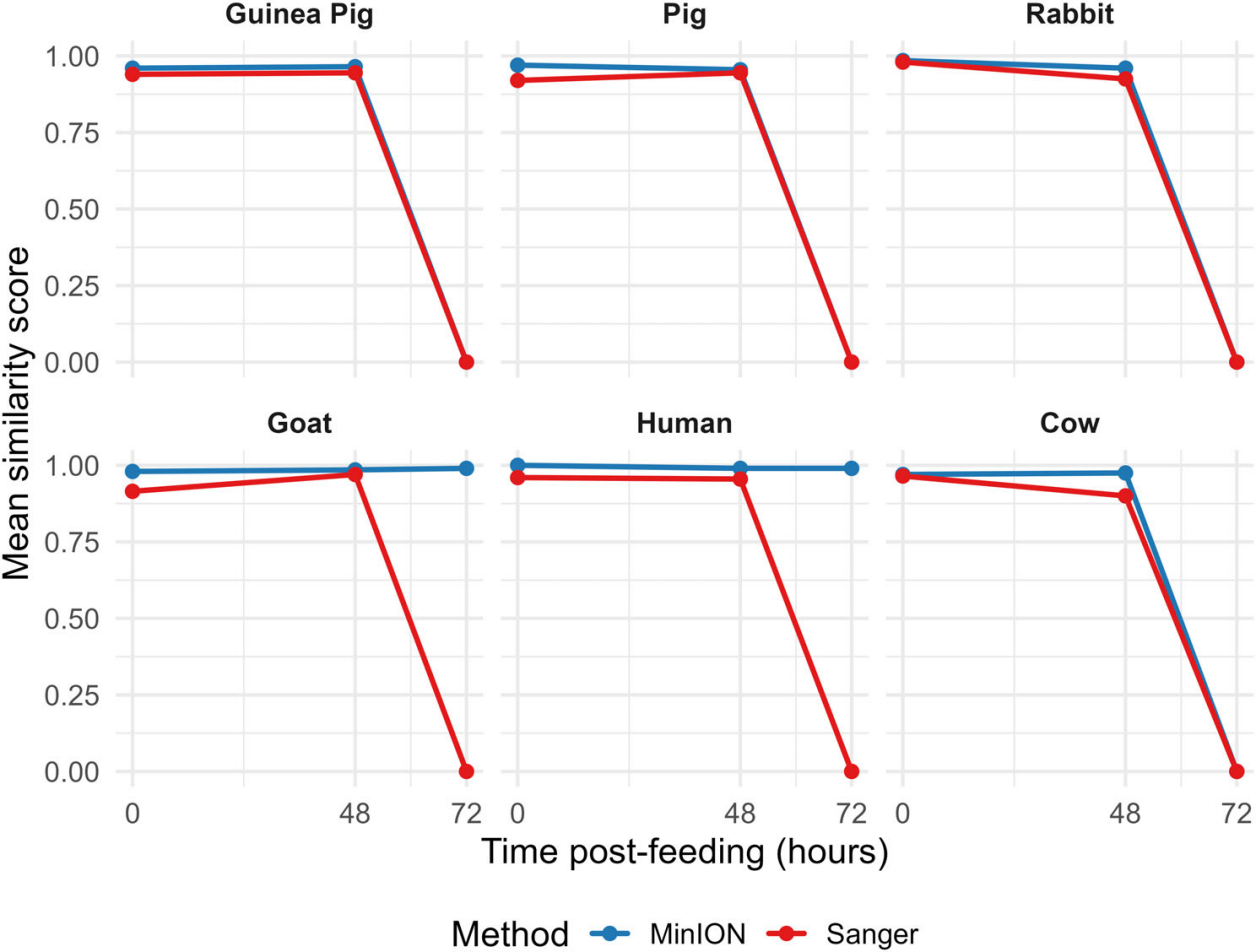
Comparison of mosquito bloodmeal host identification accuracy between Oxford Nanopore MinION and Sanger sequencing. Percent sequence similarity was calculated via BLASTn of consensus sequences for six different bloodmeal hosts at three time points post feeding. This analysis demonstrates the reliability of the MinION‐based multiplex assay in accurately identifying vertebrate bloodmeal sources over time.

Extracted DNA concentrations for these samples ranged from 16 to 127 ng/μl. Regardless of storage method, the targeted amplicon was successfully amplified using the VerU primer set. Using universal vertebrate primers, the MinION was able to detect and correctly identify the bloodmeal host from the prepared bloodspots up to 48 h post blood feeding for all host bloods used in this study. Furthermore, 50% of the samples from mosquitos fed with human or goat blood were detected 72 h after blood feeding (Figure 5). These results were consistent with all three storage methods: ethanol, DrieRite and bloodspots. Bloodmeal host was only detected for up to 48 h post feeding using Sanger sequencing with none of the host blood being detected after 48 h post blood feeding. The MinION platform detected the host in 25% more samples, with increased time post feed, showing evidence of a slightly higher sensitivity for bloodmeal detection when sequencing with the MinION compared to Sanger.

### Plasmodium *detection*

Extracted DNA concentration ranged from 54.1 to 93.1 ng/μl in the positive and negative *P. falciparum* controls. All positive controls (*N* = 20) were successfully amplified to verify the presence of *P. falciparum*. The MinION platform detected *P. falciparum* from 100% of samples that received a spiked bloodmeal of 0.5% mature gametocytes and knocked down 15 days post infectious bloodmeal. These results show an average of 98% similarity with Sanger sequencing of the same samples. The negative controls did not amplify *P. falciparum*, as expected.

## DISCUSSION

### Mosquito storage methods

We aimed to analyse samples that would be stored similarly to field‐collected specimens. For all sequencing assays, the sequence similarity scores and sequencing Q‐scores were both similar when comparing storage methods. Notably, the host bloodmeal DNA was detected with comparable read counts and coverage across all storage methods, based on qualitative assessment of sequencing output. However, it is important to note that the storage durations were not uniform across methods: samples on blood‐spotted filter paper were stored overnight, desiccant‐stored samples for 2 weeks and ethanol‐stored samples for 1 week. Because storage time is known to affect DNA degradation, these methods are not directly comparable. Nevertheless, storage in −20°C, desiccant, 100% ethanol or on blood‐spotted filter paper appears to preserve sample quality sufficiently for downstream DNA extraction.

### Simultaneous assessment

Multiplexing by utilizing the unique barcodes provided by ONT allowed for the simultaneous sequencing of several target amplicons from a single sample of extracted mosquito DNA. In this experiment, all bloodmeals were successfully detected at 48 h post feeding. Additionally, mosquitoes fed with human or goat blood showed detection in 50% of the samples at 72 h post feeding using MinION sequencing. Each of the nine represented species was also correctly identified from amplification of the ITS2 region. Lastly, 100% of the positive control *P. falciparum* samples were successfully detected, with no false positives. The negative controls did not amplify for *P. falciparum*, as expected. These results were consistent with the results generated by Sanger and MinION sequencing, confirming the status of *P. falciparum* in the samples. Simultaneous sequencing assays such as the ones described in this experiment improve the potential data output for a given sample. This enables greater time and cost efficiency when screening for multiple molecular targets as part of routine surveillance. Additionally, the MinION has previously been successfully employed in a field environment to detect other insect species (Krehenwinkel et al., 2019). As such, these methods have the potential to be successfully applied in resource‐limited field settings. The presence of 2546 unclassified reads, accounting for approximately 0.43% of the 96‐sample dataset, suggests potential index cross‐talk, which may affect the accuracy of sample assignment in future studies. This discrepancy highlights the need for careful optimization of barcoding strategies in future datasets to minimize misclassification and ensure reliable downstream analysis. However, the rapidly evolving chemistry at ONT is continuously improving the accuracy of barcode detection and reducing the likelihood of index cross‐talk. As these advancements progress, future versions of the sequencing kits and protocols may further mitigate misclassification, enhancing the reliability of sample assignments and ensuring more accurate data interpretation in subsequent sequencing runs.

Further research can apply this multiplex approach to include analysis of established single‐nucleotide polymorphisms (SNPs) that contribute to insecticide resistance, such as *kdr* and *Ace‐1* and broadened to include the detection of viruses and microbes in mosquito vectors. Further studies can also focus on the applicability of these approaches to a field setting to include rapid analyses of wild mosquitoes, parasite diversity and vector feeding habits. Additionally, the incorporation of CO1 primers for identifying bloodmeal hosts can aid in the detection of a broader range of bloodmeal hosts compared to using the vertebrate‐specific primers described in this study, providing insights into ecological dynamics and disease transmission (Mirza et al., 2024). Lastly, the approach of nanopore sequencing through adaptive sampling has been used to target the mitochondrial genome of hematophagous insects (Kipp et al., 2023) and could be further used to target specific amplicons mentioned in this study without the use of standard PCR prior to sequencing.

### MinION comparison to sanger sequencing

There are currently numerous sequencing methods with the ability to determine mosquito species, host bloodmeal source and *Plasmodium* presence. However, the MinION sequencing platform is the only device currently capable of performing all three simultaneously in a basic laboratory setting, with minimum laboratory equipment and reagent requirements. The ability to generate data in a timely manner and communicate assessments of local mosquito populations can support decision‐making in vector control strategies. Our methodology entails PCR of the three amplicons, followed by a cleanup and library preparation on the combined specimen pool, effectively simplifying the entire sequencing process with minimum training required. Additionally, reagents required for MinION sequencing, including the library preparation kit and flow cells, are stable at ambient temperature for 7 days, making it ideal for sequencing in environments where temperature control may be limited (Oxford Nanopore Technologies, 2024). Unlike Sanger sequencing, the MinION performs in real‐time and does not require a minimum read threshold for downstream bioinformatic analysis, meaning a single long read covering the entire amplicon could suffice for downstream analysis. However, for the detection of SNPs or variant detection, a minimum read count of ≥ 50 is recommended for the R9.4 flow cells, with the possibility of requiring even less reads to achieve a similar level of accuracy with the newest R10.4 chemistry (Runtuwene et al., 2018). This allows for sequencing to be halted at any time on the MinION, while also allowing for the continuance of basecalling and analysis of any data that were collected. Our results demonstrated that the MinION yielded sequencing results that were highly consistent with those from Sanger sequencing. Although the accuracy per nucleotide for the MinION platform is lower than other standard methods, such as Illumina and PacBio, regular chemistry updates continue to improve this sensitivity over time (Hamilton et al., 2023). However, the ongoing ONT updates to chemistry and software can pose challenges in establishing standard operating procedures and validation studies. Additionally, a previous study has also shown high congruence in mosquito species‐level identification between the MinION sequencer and Illumina when multiplexing mosquito bulk samples (Loh et al., 2024), suggesting a promising outlook on the use of the MinION for surveillance. Out of the 586.2 k reads obtained from the MinION sequencer for the 96 samples, 2546 were labelled as unclassified (barcode misassignment), representing approximately 0.43% of the dataset. These unclassified reads may result from index cross‐talk, where sequences are incorrectly assigned to the wrong barcode due to similar or overlapping barcode sequences, leading to misclassification (Xu et al., 2018). Unclassified reads were not considered in the analysis, and all samples were well represented.

### Data analysis

ONT provides open‐access analysis pipelines that contain user‐friendly interfaces for raw data analysis. In this study, we used ONTrack and Geneious Primer to analyse the MinION sequences. These tools were selected for their respective strengths and weaknesses to demonstrate the versatility and portability of our approach in resource‐limited field settings. Both analysis tools fully support the use of local and pre‐downloaded BLASTn databases on your local machine, allowing for offline analysis. Geneious Prime is a commercial software that, although relatively expensive, provides a user‐friendly interface for generating consensus sequences from ONT reads without requiring prior programming or bioinformatics skills. Geneious Prime allows users to select from a variety of assembly algorithms and allows for integration with external databases such as NCBI database using the Basic Local Alignment Search Tool (BLASTn). Time spent to conduct analyses on Geneious Prime varies significantly, depending on size and complexity of dataset, the number of sequences and the computational resources available. For the 96‐sample run described in this study, Geneious Prime required approximately 3 h to perform de novo assembly, alignment and BLASTn analysis of the consensus sequences. In contrast, ONTrack is a freely available software that can generate accurate consensus sequences in approximately 15 min per sample on a standard laptop. ONTrack includes an automatic BLASTn search of the consensus sequence, allowing mosquito species identification in a single pipeline from the raw ONT reads. However, using ONTrack requires familiarity with Linux command lines. Despite this, ONTrack is highly versatile for field applications as it is free, is not computationally intensive and does not require an internet connection. ONTrack is designed specifically for handling multi‐target analyses, offering more specialized support than other platforms. Nanoclust is another widely used pipeline that has shown to be excellent for clustering and identifying sequence variants but is less specialized for multi‐target analyses (Rodríguez‐Pérez et al., 2021). Along with these available pipelines, the EPI2ME software offers preconfigured workflows with cloud service capabilities, eliminating the need for dedicated bioinformaticians (Oxford Nanopore Technologies, 2024). EPI2ME is widely used in fields such as pathogen surveillance, metagenomics and epidemiological studies (Kerkhof, 2021). However, while EPI2ME is highly effective for pathogen detection and surveillance, it was not as well suited for multi‐target, multi‐species sequencing at the time of this study. The Geneious Prime and ONTrack workflows produced consistent results when aligned using BLASTn, making either method a suitable choice for analysing ONT reads. Geneious Prime offers user‐friendly features and effective visualizations of raw data, requiring no extensive bioinformatics training. However, it is more time‐consuming and necessitates purchasing a software licence. In contrast, the ONTrack pipeline, which demands moderate bioinformatics knowledge, delivers accurate consensus sequences in approximately 15 min on a standard laptop, making it a quicker alternative. This study utilizes BLASTn, which requires reliance on a pre‐curated database of known or expected pathogens and mosquito vector genomes (Boykin et al., 2019). This approach has limitations, as it may fail to identify new or emerging pathogens and vector species, particularly in the context of evolving insecticide resistance and vector surveillance. The BLASTn search of the consensus sequence against the NCBI database for taxonomic assignment could be performed both locally and online, each with its own advantages and limitations. In this study, we employed both approaches: local (ONTrack) and online BLASTn (Geneious). While the NCBI nucleotide database is known to contain uncurated and sometimes poorly annotated sequences, our approach successfully recovered the expected taxonomic assignments of the consensus sequences in all the samples, supporting its validity. This is largely due to the sufficient characterization of the *Anopheles gambiae* complex in the NCBI database. However, for field‐collected mosquitoes, BLASTn searches could be optimized by using a more streamlined database, such as a taxon‐specific subset of nt (e.g., Arthropoda or Diptera) or curated references like RefSeq, to enhance accuracy and improve computational efficiency.

### Study limitations and methodological considerations

While this study demonstrates the utility of the proposed amplicon sequencing approach for identifying mosquito species, bloodmeal sources and *Plasmodium* infection, there are several important limitations that must be acknowledged. First, the reference sequence databases for markers such as ITS2 and 16S rRNA are incomplete and often uncurated, particularly for non‐vector mosquito species. Misidentified specimens and poorly annotated sequences in public repositories such as GenBank are well documented and present a major challenge for accurate taxonomic assignment—especially outside of medically important taxa like the *Anopheles gambiae* complex (Moraes Zenker et al., 2024). The effectiveness of this method is therefore constrained by the availability and reliability of reference sequences, and no amount of database optimization can fully overcome the problem of missing or erroneous entries. ITS2, while highly informative for species identification within *Anopheles*, may be less reliable in broader taxonomic surveys. Likewise, the 16S rRNA gene offers limited species‐level resolution, and in many contexts, markers such as COI or cyt *b* may offer better coverage and discriminatory power for host identification, particularly in blood‐fed specimens collected from diverse environments (Tobe et al., 2010).

*Plasmodium* species detection also presents specific challenges. Many wildlife *Plasmodium* lineages remain uncharacterized, and public databases lack sequences for many genes across this genus. As such, there is a potential for false positives or ambiguous assignments, particularly when interpreting low‐quality or partial sequences (Slater et al., 2022). This limitation is especially important when working in ecologically complex areas with high vertebrate and parasite diversity.

Furthermore, practical considerations must be weighed when selecting a sequencing method. While the MinION platform offers high‐throughput and flexible sequencing, it may not be the most efficient approach for small‐scale studies or situations where associating data with individual mosquitoes is critical. In such cases, traditional Sanger sequencing may be more appropriate. Conversely, when processing large numbers of specimens, the labor associated with individual PCR reactions and Sanger sequencing becomes prohibitive, and a high‐throughput platform like MinION may be more suitable (Abeynayake et al., 2021). Additionally, our approach is not directly comparable to standard parasite surveillance techniques that rely on pooling mosquito samples followed by genus‐ or species‐specific PCR. Although MinION offers advantages in terms of broader taxonomic resolution and the potential to detect unexpected taxa, pooling and targeted PCR remain more practical in many surveillance settings, particularly where resources are limited or species identification is not a priority.

Overall, while our findings support the value of this approach in specific contexts, we emphasize that its utility is closely tied to the completeness and accuracy of reference databases, the ecological context of sample collection and the scale and goals of the study. Future work should aim to improve database curation, explore the performance of additional gene targets and more directly compare methodological trade‐offs in both field and laboratory settings.

## CONCLUSION

This study demonstrates the capabilities and sensitivity of the MinION sequencing platform, using a multiplex approach to detect mosquito species, bloodmeal host and *Plasmodium* infection in malaria vectors. The MinION sequencer can offer a practical and cost‐efficient addition to mosquito surveillance programmes in resource‐limited malaria endemic settings. As the technology continues to improve, increased sequencing sensitivity can be employed for more accurate genotyping of vectors with complex traits to further study the intricate interactions of vector dynamics and disease transmission.

## AUTHOR CONTRIBUTIONS

**E. Abby Rogers:** Methodology; writing – original draft; data curation; formal analysis; investigation; visualization. **Dieunel Derilus:** Formal analysis; writing – review and editing. **Christopher Sandi:** Resources. **Lisa Reimer:** Writing – review and editing; resources. **Audrey Lenhart:** Funding acquisition; conceptualization; resources; writing – review and editing. **Lucy Mackenzie Impoinvil:** Conceptualization; methodology; supervision; funding acquisition; writing – review and editing.

## FUNDING INFORMATION

This work was made possible through support from CDC's Office of Advanced Molecular Detection (AMD). This project was also supported in part by an appointment to the Research Participation Program at the Centers for Disease Control and Prevention administered by the Oak Ridge Institute for Science and Education through an interagency agreement between the U.S. Department of Energy and the Centers for Disease Control and Prevention.

## CONFLICT OF INTEREST STATEMENT

LJR is on the editorial board of Medical and Veterinary Entomology; however, they played no role in the assessment of the manuscript. All other authors declare no conflicts of interest.

## CONSENT FOR PUBLICATION

All authors consent to publication.

## Supporting information


**Table S1.** Input DNA concentration and sequencing output metrics for each of the 96 barcoded samples processed in a single MinION run. For each sample, the table includes the input concentration (ng/μL), total read count and read length range (bp) following basecalling and demultiplexing.
**Figure S1.** Agarose gel image of the targeted amplicon produced when ITS2 primers amplified DNA from *Anopheles*. **Lane 1**: 1 kb Ladder, **Lanes 2–6**: *Anopheles gambiae* (expected band size = ~567 bp), **Lanes 7–12**: *Anopheles stephensi* (expected band size = ~569 bp), **Lanes 13–17**: *Anopheles minimus* (expected band size = ~560 bp).
**Figure S2.** Agarose gel image of the targeted amplicon produced when VerU primers amplified DNA from rabbit blood‐fed samples of *Anopheles albimanus* (Sanarate) (expected band size = ~280 bp). These samples were stored in DrieRite desiccant prior to DNA extraction. The g gel reads as follows: **Lane 1**: 1 kb Ladder, **Lanes 2–4**: Specimens 0 h post bloodfeed, **Lanes 5–7**: Specimens 24 h post bloodfeed **Lanes 8–10**: Specimens 48 h post bloodfeed, Lanes **11–13:** Specimens 72 h post bloodfeed, **Lane 14**: Negative Control.
**Figure S3.** The SSU_Plas primer set successfully amplified *Plasmodium* in 100% of the positive control samples. Expected band size for the SSU_Plas PCR product is 422 bp. **Lane 1:** 1 kb ladder, **Lanes 2–10:** Positive control specimens that had been fed spiked bloodmeal of 0.5% mature gametocyte, **Lane 11:** Negative control.
**Figure S4.** Flowchart describing the bioinformatics workflow using the ONTrack pipeline adapted from Maestri et al., 2019.

## Data Availability

All sequence data generated during the course of this study have been deposited in public repositories and are publicly available. The data are accessible via Dryad at https://doi.org/10.5061/dryad.c59zw3rp8. Consensus sequences generated from Oxford Nanopore amplicon sequencing have been deposited in NCBI GenBank under accession numbers PV031619.1‐PV031627.1, PX684277‐PX684280 and PV031041.1. Reference sequences used for mosquito species, bloodmeal host and *Plasmodium* identification were obtained from publicly available NCBI GenBank records.
