# Supplementary material for: A multiplex assay to detect mosquito species, bloodmeal host source and Plasmodium in malaria vectors using Nanopore amplicon sequencing
Source: Med Vet Entomol. 2026 Feb 12;40(3):465–77. doi: 10.1111/mve.70055 (PMC13436545; doi:10.1111/mve.70055)
Supplement: Supplementary file 1 [file MVE-40-465-s001.docx]

Supplementary Figures

**Table S1.**

Input DNA concentration and sequencing output metrics for each of the 96 barcoded samples processed in a single MinION run. For each sample, the table includes the input concentration (ng/µL), total read count and read length range (bp) following basecalling and demultiplexing.

| **Barcode** | **Target** | **Product** | **Total Sequence Reads** | **Sequence Length** | **Sample Concentration** |
| --- | --- | --- | --- | --- | --- |
| 1 | ITS2 | *An. stephensi* | 3092 | 154-740 | 72.7 |
| 2 | ITS2 | *An. coluzzi* | 3270 | 149-727 | 65.1 |
| 3 | ITS2 | *An. minimus* | 1588 | 162-677 | 49.4 |
| 4 | ITS2 | *An. coluzzi* | 3826 | 138-1297 | 78.4 |
| 5 | ITS2 | *An. funestus* | 4088 | 153-591 | 82.2 |
| 6 | ITS2 | *An. albimanus* | 19966 | 141-1058 | 112 |
| 7 | ITS2 | *An. albimanus* | 1452 | 172-657 | 15.6 |
| 8 | ITS2 | *An. gambiae* | 1648 | 157-1093 | 18.4 |
| 9 | ITS2 | *An. arabiensis* | 300 | 140-723 | 9.2 |
| 10 | ITS2 | *An. merus* | 780 | 159-679 | 23.7 |
| 11 | ITS2 | *An. freeborni* | 1930 | 159-665 | 36.1 |
| 12 | ITS2 | *An. albimanus* | 1422 | 166-638 | 47.6 |
| 13 | ITS2 | *An. funestus* | 522 | 156-696 | 14.2 |
| 14 | ITS2 | *An. minimus* | 12840 | 135-1287 | 75.3 |
| 15 | ITS2 | *An. merus* | 4914 | 148-638 | 66.3 |
| 16 | ITS2 | *An. arabiensis* | 1728 | 105-832 | 158.2 |
| 17 | ITS2 | *An. albimanus* | 4556 | 138-747 | 78.4 |
| 18 | ITS2 | *An. gambiae* | 2294 | 153-810 | 63.9 |
| 19 | ITS2 | *An. arabiensis* | 850 | 88-810 | 23.2 |
| 20 | ITS2 | *An. stephensi* | 2696 | 135-726 | 55.5 |
| 21 | ITS2 | *An. coluzzi* | 1052 | 159-713 | 28.7 |
| 22 | ITS2 | *An. arabiensis* | 1922 | 147-693 | 32.1 |
| 23 | ITS2 | *An. freeborni* | 146 | 174-846 | 6.5 |
| 24 | ITS2 | *An. minimus* | 1376 | 151-743 | 17.3 |
| 25 | ITS2 | *An. freeborni* | 4516 | 152-668 | 69.4 |
| 26 | ITS2 | *An. gambiae* | 13018 | 128-651 | 98.4 |
| 27 | ITS2 | *An. funestus* | 5018 | 146-660 | 83.9 |
| 28 | ITS2 | *An. coluzzi* | 1284 | 155-545 | 26.7 |
| 29 | ITS2 | *An. albimanus* | 9988 | 133-783 | 72.7 |
| 30 | ITS2 | *An. merus* | 15024 | 148-653 | 86.5 |
| 31 | ITS2 | *An. stephensi* | 1290 | 135-733 | 52.8 |
| 32 | ITS2 | *An. coluzzi* | 3908 | 169-870 | 67.7 |
| 33 | ITS2 | *An. freeborni* | 1950 | 150-675 | 26.4 |
| 34 | SSU_Plas | *P. falciparum* | 7920 | 159-452 | 71.2 |
| 35 | SSU_Plas | *P. falciparum* | 1014 | 167-569 | 56.3 |
| 36 | SSU_Plas | *P. falciparum* | 14920 | 138-510 | 47.8 |
| 37 | SSU_Plas | *P. falciparum* | 14146 | 151-423 | 77.6 |
| 38 | SSU_Plas | *P. falciparum* | 2060 | 168-659 | 63.3 |
| 39 | SSU_Plas | *P. falciparum* | 2586 | 158-789 | 51.9 |
| 40 | SSU_Plas | *P. falciparum* | 1302 | 163-487 | 15.9 |
| 41 | SSU_Plas | *P. falciparum* | 4420 | 155-784 | 33.7 |
| 42 | SSU_Plas | *P. falciparum* | 5826 | 113-467 | 56.5 |
| 43 | SSU_Plas | *P. falciparum* | 8212 | 170-682 | 96.7 |
| 44 | SSU_Plas | *P. falciparum* | 3288 | 152-443 | 22.6 |
| 45 | SSU_Plas | *P. falciparum* | 1374 | 146-511 | 31.1 |
| 46 | SSU_Plas | *P. falciparum* | 14322 | 137-401 | 86.1 |
| 47 | SSU_Plas | *P. falciparum* | 12186 | 149-528 | 77.7 |
| 48 | SSU_Plas | *P. falciparum* | 17806 | 156-648 | 141.6 |
| 49 | SSU_Plas | *P. falciparum* | 5036 | 142-684 | 63.6 |
| 50 | SSU_Plas | *P. falciparum* | 25196 | 184-502 | 99.6 |
| 51 | SSU_Plas | *P. falciparum* | 25736 | 148-356 | 87.5 |
| 52 | SSU_Plas | *P. falciparum* | 12420 | 149-691 | 66.8 |
| 53 | SSU_Plas | *P. falciparum* | 11616 | 148-694 | 54.2 |
| 54 | SSU_Plas | *P. falciparum* | 9584 | 103-541 | 49.9 |
| 55 | SSU_Plas | *P. falciparum* | 2266 | 137-628 | 42.7 |
| 56 | SSU_Plas | *P. falciparum* | 10716 | 142-669 | 63.3 |
| 57 | SSU_Plas | *P. falciparum* | 3910 | 190-367 | 36.4 |
| 58 | SSU_Plas | *P. falciparum* | 13192 | 143-477 | 77.1 |
| 59 | SSU_Plas | *P. falciparum* | 8788 | 206-615 | 12.3 |
| 60 | SSU_Plas | *P. falciparum* | 2244 | 154-675 | 26.9 |
| 61 | SSU_Plas | *P. falciparum* | 1722 | 151-400 | 15.8 |
| 62 | SSU_Plas | *P. falciparum* | 1836 | 155-461 | 36.1 |
| 63 | SSU_Plas | *P. falciparum* | 6324 | 121-708 | 68.2 |
| 64 | SSU_Plas | *P. falciparum* | 2844 | 160-351 | 51.1 |
| 65 | SSU_Plas | *P. falciparum* | 3402 | 106-399 | 32.6 |
| 66 | SSU_Plas | *P. falciparum* | 23532 | 135-297 | 98.4 |
| 67 | VerU | Cow (0hrs) | 13708 | 148-682 | 72.4 |
| 68 | VerU | Cow (0hrs) | 8262 | 176-460 | 68.8 |
| 69 | VerU | Cow (48hrs) | 2334 | 143-704 | 42.1 |
| 70 | VerU | Cow (48hrs) | 4120 | 136-489 | 23.9 |
| 71 | VerU | Cow (72hrs) | 5722 | 172-682 | 26.1 |
| 72 | VerU | Cow (72hrs) | 4898 | 156-930 | 15.9 |
| 73 | VerU | Guinea Pig (0hrs) | 4976 | 151-420 | 25.8 |
| 74 | VerU | Guinea Pig (0hrs) | 184 | 158-588 | 33.9 |
| 75 | VerU | Guinea Pig (48hrs) | 7378 | 134-454 | 56.7 |
| 76 | VerU | Guinea Pig (48hrs) | 1690 | 132-422 | 12.3 |
| 77 | VerU | Guinea Pig (72hrs) | 4760 | 50-117 | 15.7 |
| 78 | VerU | Guinea Pig (72hrs) | 7874 | 13-65 | 4.6 |
| 79 | VerU | Goat (0hrs) | 14622 | 142-520 | 41.5 |
| 80 | VerU | Goat (0hrs) | 1042 | 167-422 | 5.8 |
| 81 | VerU | Goat (48hrs) | 1176 | 137-418 | 8.4 |
| 82 | VerU | Goat (48hrs) | 11718 | 127-413 | 36.4 |
| 83 | VerU | Goat (72hrs) | 4786 | 128-463 | 23.9 |
| 84 | VerU | Goat (72hrs) | 6220 | 186-420 | 35.5 |
| 85 | VerU | Rabbit (0hrs) | 3452 | 144-422 | 45.3 |
| 86 | VerU | Rabbit (0hrs) | 12304 | 143-511 | 87.2 |
| 87 | VerU | Rabbit (48hrs) | 1866 | 147-486 | 16.3 |
| 88 | VerU | Rabbit (48hrs) | 11624 | 146-431 | 56.8 |
| 89 | VerU | Rabbit (72hrs) | 218 | 6-13 | 13.1 |
| 90 | VerU | Rabbit (72hrs) | 378 | 14-51 | 6.4 |
| 91 | VerU | Human (0hrs) | 2858 | 168-883 | 12.7 |
| 92 | VerU | Human (0hrs) | 956 | 130-865 | 15 |
| 93 | VerU | Human (48hrs) | 2218 | 130-713 | 21.1 |
| 94 | VerU | Human (48hrs) | 12862 | 140-740 | 75.4 |
| 95 | VerU | Human (72hrs) | 6356 | 123-177 | 63.3 |
| 96 | VerU | Human (72hrs) | 7720 | 162-212 | 48.2 |
| Unclassified | - | - | 2546 | - | - |
| **Total** | **-** | **-** | **586828** | **-** | **-** |

Fig. S1.

Agarose gel image of the targeted amplicon produced when ITS2 primers amplified DNA from *Anopheles*. **Lane 1**: 1kb Ladder, **Lanes 2-6**: *Anopheles gambiae* (expected band size = ~567bp), **Lanes 7-12**: *Anopheles stephensi* (expected band size = ~569bp), **Lanes 13-17**: *Anopheles minimus* (expected band size = ~560bp).

Fig. S2.

Agarose gel image of the targeted amplicon produced when VerU primers amplified DNA from rabbit bloodfed samples of *Anopheles albimanus* (Sanarate) (expected band size = ~280bp). These samples were stored in DrieRite dessicant prior to DNA extraction. The g gel reads as follows: **Lane 1**: 1kb Ladder, **Lanes 2-4**: Specimens 0 hours post bloodfeed, **Lanes 5-7**: Specimens 24 hours post bloodfeed **Lanes 8-10**: Specimens 48 hours post bloodfeed, Lanes **11-13:** Specimens 72 hours post bloodfeed, **Lane 14**: Negative Control

Fig. S3.

The SSU_Plas primer set successfully amplified *Plasmodium* in 100% of the positive control samples. Expected band size for the SSU_Plas PCR product is 422bp. **Lane 1:** 1 kb ladder, **Lanes 2-10:** Positive control specimens that had been fed spiked bloodmeal of 0.5% mature gametocyte, **Lane 11:** Negative control.

Fig. S4.

Flowchart describing the bioinformatics workflow using the ONTrack pipeline adapted from Maestri et al., 2019.
